# Gender differences in Myasthenia Gravis in a neuromuscular reference center in Belgium

**DOI:** 10.1007/s13760-025-02977-8

**Published:** 2026-01-05

**Authors:** Alicia Alonso-Jiménez, Willem De Ridder, Paul Van Schil, Jonathan Baets, Rudy Mercelis

**Affiliations:** 1 Department of Neurology, Antwerp University Hospital, Antwerp, Belgium; 2 Institute Born-Bunge and Translational Neurosciences, Faculty of Medicine and Health Sciences,, University of Antwerp, Antwerp, Belgium; 3 Department of Thoracic and Vascular Surgery, Antwerp University Hospital, University of Antwerp, Antwerp, Belgium

**Keywords:** Gender differences, Myasthenia Gravis

## Abstract

**Background:**

It is well known that scientific literature has a significant gender gap, as gender differences were not considered until very recently. Women were often excluded from studies, and even when included, the results are rarely analyzed separately by gender. This study aims to investigate gender-specific differences in patients with Myasthenia Gravis (MG) by segregating data from our cohort of patients in Antwerp (Belgium).

**Methods:**

We analyzed the data of our previously published cohort of 163 patients with MG visited in the Antwerp University Hospital between 2019 and 2021, segregating the information by gender to observe any significant differences.

**Results:**

The analysis revealed several notable gender-specific differences. Women experienced a delay in diagnosis of over one year more frequently than men. They also had dysarthria as presenting symptom more often than men. The MGFA scores at maximum severity were higher in women, who also reported more limitations due to the disease and required more treatments to control it. While some differences could be attributed to the younger onset of the disease in women, certain differences were independently influenced by gender.

**Conclusions:**

Women experience more limitations due to MG than men and may face a more severe disease course. These differences should be taken into account when determining follow-up and treatment strategies. Additionally, these findings highlight the importance of segregating data by gender in scientific studies to better understand gender-specific differences in disease presentation and management.

## Introduction

Myasthenia Gravis (MG) is an autoimmune neuromuscular disorder characterized by muscle weakness and fatigue. Although it has classically been stated that MG is more frequent in women than men, recent studies confirm this difference only in early-onset MG, while in the late-onset form both genders have equal frequency, or it could even be more frequent in men [1–4].

Although extensive research has shown remarkable pathophysiological differences between genders [1], and other works have drawn attention to the gender gap in medicine [5–8], current research studies continue to not report the results segregated by gender.

In MG in particular, there are many articles describing cohorts of patients, but apart from mentioning the well-known difference in age of onset, the articles that separated their results by gender are still a minority [9–12]. However, these scarce literature shows differences in severity of the disease and quality of life, with women being more affected than men [9, 10, 13–19].

Because the immune response, the response to treatment and the incidence of adverse events between women and men could differ, we decided to extract our results from our previously published cohort of patients and separate them by gender.

## Methods

### Patients

Patient data were retrospectively retrieved from our database. We included patients who had a neurology consultation during 2019, 2020, or 2021 with a diagnosis of MG. Further details regarding patient selection have been reported previously [20].

### Statistics

Results are reported as numbers and percentages. Differences in binary variables between men and women were analysed with Chi-square test or Fisher exact test when numbers are small. For ordinal data Mann-Whitney U test was used for comparison between men and women.

As age at diagnosis is known to be considerably lower in women compared to men, we used logistic regression to correct for the differences in age at diagnosis when looking at binary outcomes. For ordinal outcomes a proportional odds ordinal logistic regression model was used. All models included sex and age at diagnosis as independent variables.

For all statistical analyses and graphs, we used IBM^®^ SPSS^®^ Statistics version 27. A p-value below 0.05 was considered significant.

## Results

### Patients

We included 163 patients. 86 (52.8%) were women and 77 were men (47.2%). Sex and age at diagnosis were reported in our previous article [20], and it showed two peaks of incidence for women (between 20 and 40 years and above 60) and only one peak for men (above 50 years).

### Serological status

Antibodies against AChR were positive in 135/162 patients (83.3%). By gender, 68/85 women (80%) and 67/77 men (87%) were positive, with no significant difference (*p* = 0.23). Anti-MuSK antibodies were tested in 26 patients: 12 AChR-positive and 14 AChR-negative. Among them, 6 were anti-MuSK positive (5/17 women and 1/9 men, *p* = 0.29). No patients were double-positive for both AChR and MuSK.

Anti-LRP4 antibodies were tested in 6 patients and were negative in all cases (4 women, 2 men). Among the 27 AChR-negative patients, anti-MuSK testing was not performed in 13 (7 men, 6 women). Eight patients were double seronegative (2 men, 6 women). When grouping the single seronegative patients (AChR negative, not tested for MuSK) together with the double seronegative patients, no significant gender difference was observed (*p* = 0.64).

### First symptoms

**Fig. 1:**
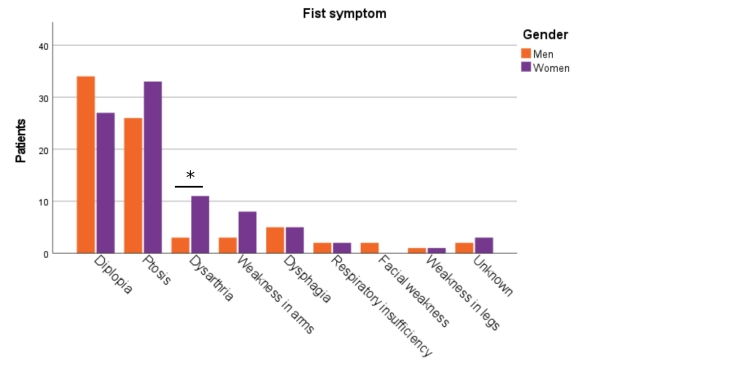
First symptom of Myasthenia Gravis of the patients of the study, segregated by gender

Regarding the first symptom, ptosis and diplopia were the most frequent in both genders (Fig. 1). Five patients (4 women and 1 men) referred both ptosis and diplopia as the presenting symptom. For clarity, both symptoms have been counted apart in the graph. Dysarthria was significantly more frequent in women than in men (11/83 [13.3%] vs. 3/75 [4%], *p* = 0.041). Weakness in the upper limbs was more frequent in women (8/83 [9.6%] vs. 3/75 [4%]), showing a tendency without reaching statistical significance.

### Evolution

**Fig. 2:**
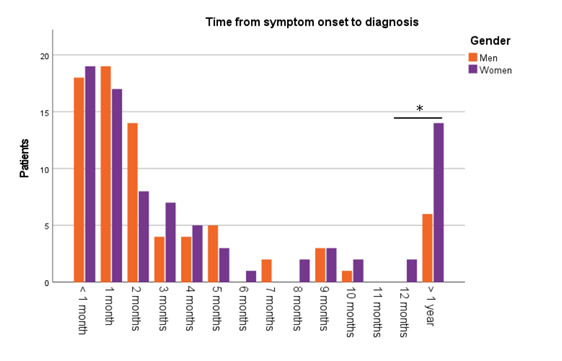
Time from onset of symptoms to diagnosis of Myasthenia Gravis, segregated by gender. Delayed diagnoses > 11 months were significantly more frequent in women than in men (*p* = 0.038, indicated by *)

Time from symptom onset to diagnosis was similar in both genders (*p*=0.19), with a median interval of 2 months. However, delayed diagnoses beyond 11 months occurred significantly more often in women than in men (*p*=0.038, Figure 2).

Regarding the follow-up time of our patients, women were followed-up for a significant longer period of time (median for men = 62 months, women = 88,5 months, *p* = 0.04), although there was a wide spread in the data (SD = 68,78 for men and 129,15 for women).

We were interested in knowing whether this longer follow-up period could have an impact in the number of women reaching their maximun severity statusWe compared the time (in months) from diagnosis to maximun severity, excluding patients who only had ocular symptoms during the whole follow-up. The median time was 3 months for men and 10 months for women. Data was also highly dispersed and the difference between genders was not statistically significant (*p* = 0.16).

### Myasthenia Gravis foundation of America (MGFA) clinical classification

**Fig. 3:**
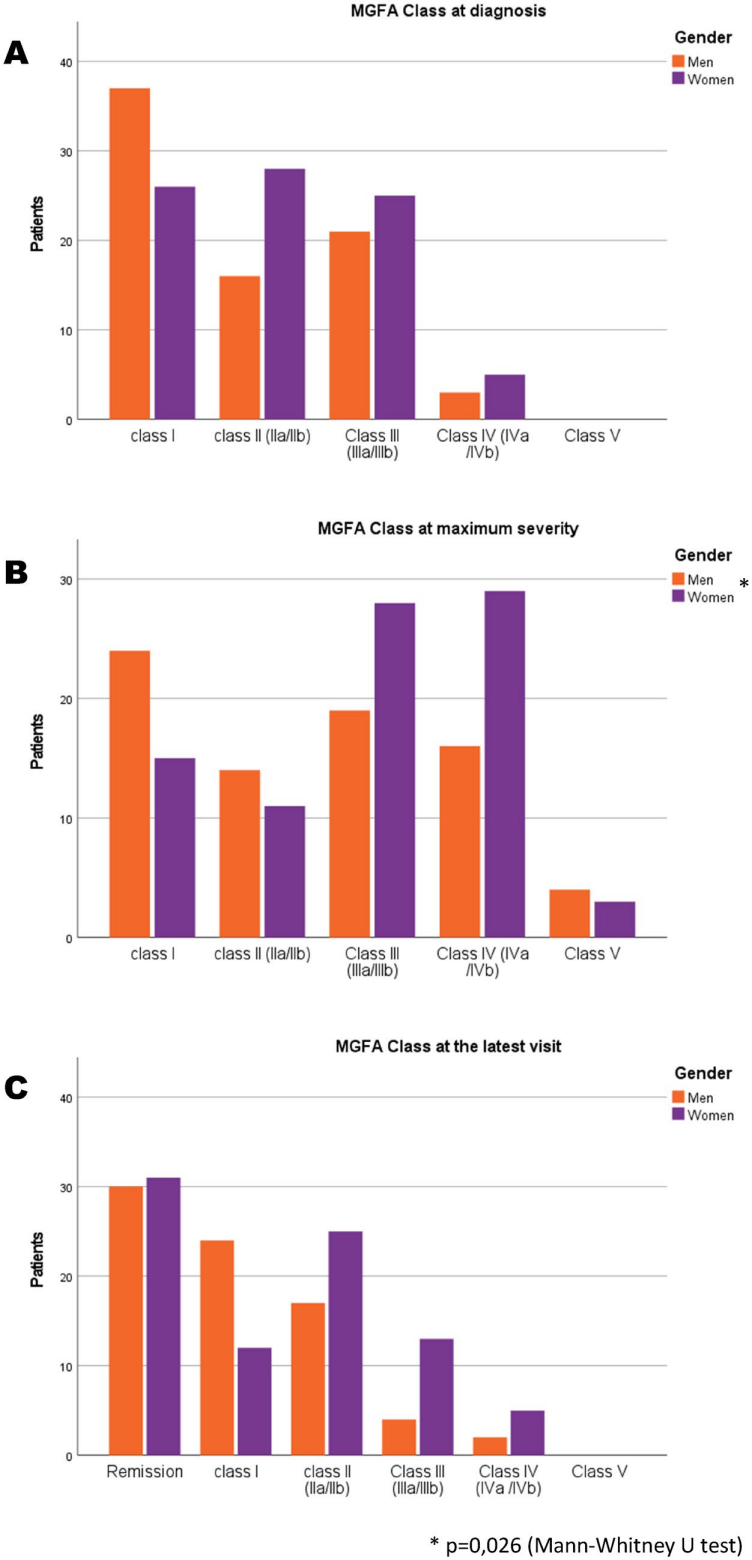
Myasthenia Gravis Foundation of America (MGFA) clinical classification at the diagnosis (**A**), at maximum severity of the disease (**B**) at the latest visit (**C**), segregated by gender. Statistical significant differences are indicated with *

At the time of diagnosis, the majority of men belonged to MGFA class I (ocular MG [oMG]), while most women were class II, followed closely by class I (Fig. 3A). These differences were not statistically significant (Mann-Whitney U test *p* = 0.092).

At maximum severity, however, the differences did reach statistical significance: while the majority of men were still class I, followed by class III and IV, the majority of women reached class IV, followed closely by class III (Mann-Whitney U test *p* = 0.026, Fig. 3B). When we corrected for age at diagnosis with a ordinal logistic regression (to check whether the differences were due to the gender or to the age at diagnosis), we still found an independent influence of gender (*p* = 0.048).

We did not find any differences between men and women regarding the time from diagnosis to maximum severity (*p* = 0.28).

At the latest visit, the majority of patients were in remission for both genders. However, while the second most frequent status for men was MGFA class I, for women it was class II followed by class III (Mann-Whitney U test *p* = 0.57, Fig. 3C).

### MGFA Post-intervention status (MGFA-PIS)

**Fig. 4:**
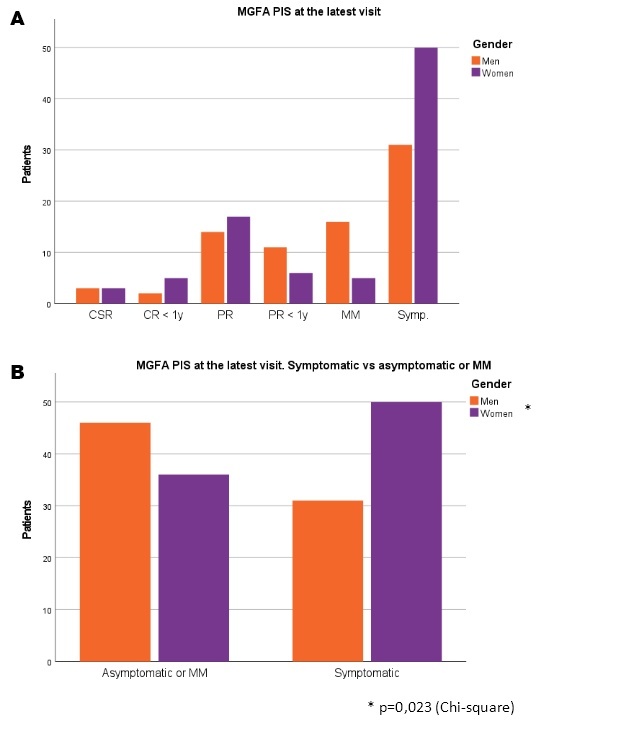
Myasthenia Gravis Foundation of America Post-intervention Status (MGFA-PIS). **A** distribution of patients according to the MGFA-PIS categories. **B** Distribution of patients grouped based on whether they experience limitations due to the disease (symptomatic) or not (asymptomatic or minimal manifestations). CSR: complete stable remission. CR < 1y: patients without symptoms or signs who discontinued medication less than one year before the last visit and therefore do not meet the criteria for CSR. PR: Pharmacological Remission. PR < 1y: pharmacological remission for less than a year. MM: Minimal manifestations. Symp: Symptomatic patients who do not fit into any of the previous categories

The number of women in complete or pharmacological remission (including those that were in remission for less than a year and therefore not fulfilling the criteria for remission according to MGFA Post-intervention Status [MGFA-PIS]) at the latest visit was 31 (36% of total women). For men it was 30 (39%). In addition, 5 women (6%) and 16 men (21%) had minimal manifestations according to the MGFA-PIS. Therefore, a total of 36 women (42%) and 46 men (60%) had no significant limitations due to MG. 50 women (58%) and 31 men (40%) were still symptomatic at the latest visit (Fig. 4A).

If we divide the patients into two groups, those who experience limitations from the disease and those who are in remission or with minimal manifestations according to the MGFA-PIS, statistically significant differences were found between men and women (Chi-square, *p*=0.023, figure), women being more frequently symptomatic (Figure 4B).

When we apply logistic regression analysis controlling for age at diagnosis, the differences are borderline not significant anymore (*p* = 0.073), indicating that age of onset of the disease also plays a role.

### Treatment

Regarding the intake of pyridostigmine, most patients had taken it at least at some point in the course of their disease, without significant differences between genders (83 women [96.5%] and 74 men [96.1%]).

No differences were present either in the rescue therapies with plasmapheresis or intravenous immunoglobulins. Twenty-seven women (31.4%) and 23 men (29.9%) were treated with these therapies at some point in the course of their disease.

**Fig. 5:**
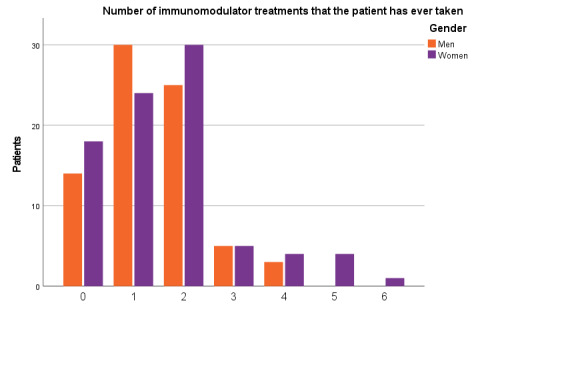
Amount of immunomodulator treatments that the patients had ever taken, segregated by gender. Pyridostigmine, plasmapheresis and immunoglobulins are not included

Regarding the number of immunomodulator treatments that both genders had taken, the majority of men had taken just one (30 men, 39%), while most women had taken two (30 women, 34.9%). Remarkably, 4 women had taken 5 immunotherapies, and one woman even 6, while no man in our cohort took more than 4 (Fig. 5). These differences were not significant (*p* = 0.39). Treatments included azathioprine, corticosteroids (prednisolone or methylprednisolone), mycophenolate mofetil, cyclosporine, tacrolimus, methotrexate, rituximab, eculizumab, and efgartigimod; the latter two were administered to two patients within clinical trials. Details on treatment distribution have been reported previously [20].

Regarding surgery, 41 women (47.7%) and 17 men (22.1%) underwent thymectomy (*p* < 0.001). Therefore, this procedure was more frequently performed in women. When we corrected by age at diagnosis with a logistic regression, we still found that sex had an independent relation with the procedure (*P* = 0.036).

Thirteen women (15.1%) and 8 men (10.4%) had a thymoma. Regarding the type of thymoma, among the men, there were 3 type AB, 2 type B1, 2 type B2, 1 type B2-B3 and one benign mesothelial cyst. Among women, 2 type A, 1 type A-B, 5 type B2, 2 type B2-B3, 1 type B3, 1 type B4, 2 thymolipomes and one epithelial and lymphoid thymoma.

Thymus hyperplasia was present in 6 men and 14 women. In 6 women was the result of the pathology not known.

## Discussion

In our cohort of 163 patients with MG, we observed significant gender-related differences in disease evolution and severity. Specifically, women more frequently presented with dysarthria as their first symptom, experienced diagnostic delays beyond 11 months, reached higher MGFA scores at maximum severity, and underwent thymectomy more often than men.Previous studies have reported gender-based differences in patients with MG. Not only is the incidence higher in young women, but they seem to have therapy resistant disease more frequent than males [13, 14]. Furthermore, a recent meta-analysis of thirty-one studies suggested that females are more likely to evolve from oMG to generalized MG (gMG) [21]. Some articles have shown that women score worse than men in self-reported scores about disease severity (Myasthenia Gravis Activities of Daily Living [MG-ADL] score), fatigue sum scores and quality of life scores (MG-QOL15, Short Form Health Survey 36 [SF-36]) [9, 10, 15–19]. Particularly interesting is the prospective study of Thomsen et al. [10], which shows that women improved less than men on the quantitative MG test (QMG), MG composite (MGC), MG-ADL and QOL15 independent of MG duration, patient age or presence or not of refractory disease.

The reasons for these differences are poorly understood. Younger onset in women has been pointed out, but as the prospective study above-mentioned shows, female gender seems to be an independent factor. Our study also supports this assumption. Other possibility that has been addressed is possible differences in treatment approach between females and males. Women are thought to refuse more frequently corticosteroids due to adverse events, however, there is little literature that supports this belief [22]. Suresh et al. [11] reported differences in CT chest screening and pharmacological treatment between males and females in a large cohort in the United States. However, Beland et al. [12] did not find differences in treatment or timing of treatment in gMG in Canada. Therefore, it is not clear whether differences in care play a role. Dong et al. [18] found that the presence of comorbidities had a bigger impact in the QoL in females than men. The explanation for the differences between genders it’s probably multifactorial, and social, physical or emotional factors may play a role that it is difficult to assess.

Unfortunately, one of the limitations of our study is that we did not collect MG-ADL scores nor quality of life scales, so we can not compare our results with the above-mentioned studies. However, we did see differences in initial symptoms, treatments and evolution of the disease measured with the MGFA class. Regarding the first manifestations, the most frequent presenting symptoms in both genders were ocular. However, dysarthria and upper limb weakness were more common as presenting symptoms in women. This highlights the importance of including MG in the differential diagnosis when women present with these specific symptoms.

Other studies have mention a longer delay in the diagnosis of MG in female patients [9, 15, 23], so we were especially interested in checking if this was also true for our cohort. Time from onset of symptoms to diagnosis was similar in both genders, being the median interval 2 months, but there was a noticeable difference: a delayed diagnosis above one year was far more frequent in women than men. Although this accounted only for a small number of patients, it’s important to report it.

Regarding the severity of the disease measured by the MGFA class, most men had only ocular symptoms at the time of diagnosis (MGFA Class I) while most women had already generalized involvement (MGFA Class II), although the differences were not statistically significant. However, at maximum severity, the differences were more pronounced: while the majority of men were still in class I, most women reached class IV. These differences were significant even when we corrected by age at diagnosis, indicating that women experience at some point in the evolution of their MG more severe symptoms. The longer follow-up time in women could also play a role, but in our cohort there were no significant differences between genders in the time from diagnosis to maximum severity, so we think that this influence is probably limited. Fortunately, in the last visit in our clinic, most patients were in remission for both genders. However, when we compared patients with minimal manifestations or better with symptomatic patients, there were more symptomatic women than men. Correcting by age we found that these differences were indeed partly influenced by the age at diagnosis, underlining the importance of the incidence peak of MG in young women.

It makes sense that, if women get diagnosed at a younger age than men, and therefore have a longer disease course with more severe symptoms like our study shows, they will receive more treatments. This is also suggested by our study: while no men received more than 4 immunosuppressive treatments, a small number of women received up to 6. This was true despite thymectomy being more frequently performed in women, which is known to be associated with better outcomes, less myasthenic crisis and less need for medication [24, 25]. Unfortunately, data on the reasons for treatment discontinuation were not systematically collected. Among the five women who received ≥ 5 therapies, three escalated due to lack of efficacy, one due to adverse events, and one due to a combination of both. All had gMG (three anti-AChR positive, two anti-MuSK positive), and both anti-MuSK positive patients ultimately remained on rituximab.

Our study has several limitations. First, it is a retrospective analysis based on a small, single-center cohort, which may limit the generalizability of our findings. Second, we did not collect standardized Myasthenia Gravis (MG) severity scores, such as MG-ADL, nor did we assess quality of life measures. Additionally, the data collection was conducted during the COVID-19 pandemic, which may have discouraged asymptomatic or mildly symptomatic patients from seeking medical attention, potentially introducing a selection bias toward more severe cases.

In conclusion, the gender-segregated data from our study supports the evidence that the evolution of MG differs between men and women, with women experiencing a more severe disease course and requiring more medication. This difference is partially, but not entirely, explained due to the earlier onset of the disease in women. Hormonal or socioeconomical factors may also contribute, which would require further research. These differences should be considered in clinical practice and research on MG. Our study also underscores the importance of reporting results segregated by gender.

## Data Availability

The data processed in this paper are all available upon request.
